# Influenza A(H5N2) Virus Antibodies in Humans after Contact with Infected Poultry, Taiwan, 2012

**DOI:** 10.3201/eid2005.131393

**Published:** 2014-05

**Authors:** Ho-Sheng Wu, Ji-Rong Yang, Ming-Tsan Liu, Chin-Hui Yang, Ming-Chu Cheng, Feng-Yee Chang

**Affiliations:** Centers for Disease Control, Taipei, Taiwan (H.-S. Wu, J.-R. Yang, M.-T. Liu, C.-H. Yang, F.-Y. Chang);; Taipei Medical University, Taipei (H.-S. Wu);; Animal Health Research Institute, Taipei (M.-C. Cheng);; National Defence Medical Center, Taipei (F.-Y. Chang)

**Keywords:** HPAI H5N2 virus, NS1 antibody, seroconversion, influenza, respiratory infections, poultry, antibodies, influenza A(H5N2) virus, viruses, humans, cross-reactivity, contact, Taiwan

## Abstract

Six persons in Taiwan who had contact with poultry infected with influenza A(H5N2) showed seroconversion for the virus by hemagglutinin inhibition or microneutralization testing. We developed an ELISA based on nonstructural protein 1 of the virus to differentiate natural infection from cross-reactivity after vaccination; 2 persons also showed seroconversion by this test.

Since 1959, highly pathogenic avian influenza A (HPAI) subtypes H5 and H7 have caused outbreaks in poultry resulting in high mortality rates and have also caused sporadic infections in humans (*1*–*3*). Some low pathogenicity avian influenza (LPAI) viruses can mutate to become HPAI virus by acquiring basic amino acid residues in the hemagglutinin (HA) cleavage site after multiple passages in chickens (*4*). In Taiwan, poultry infected by LPAI (H5N2) virus were reported during 2003–2004 and 2008–2011 (*5*–*7*), whereas HPAI (H5N2) viruses were first isolated in 2012 and caused subsequent outbreaks in poultry. Although >20 differences have been found in amino acids in the HA protein between the 2012 HPAI (H5N2) viruses and the 2003 LPAI (H5N2) virus (A/chicken/Taiwan/1209/2003), these viruses are antigenically similar (M.-C. Cheng, unpub. data) and related to those that circulated in Mexico in 1994 but unrelated to the subtype H5N1 viruses that reemerged in 2003 and the subtype H5N1 vaccine strain (A/Vietnam/1194/2004) (*5*).

As of December 23, 2013, influenza A(H5N2) virus had not been isolated from humans, but previous studies have provided serologic evidence for subclinical infections in persons who had frequent contacts with infected animals (*3*,*8*–*11*). We therefore investigated the possibility of infection among persons who were exposed to HPAI (H5N2) virus during outbreaks in chicken farms in Taiwan during January–March 2012.

## The Study

For our study, we enrolled 141 persons who had close contact with poultry at 5 chicken farms that had influenza A(H5N2) outbreaks in chickens during January–March 2012. These contacts were 15 farm workers, 90 animal health officials, and 36 temporary employees who participated in culling of infected chickens; no symptoms of influenza-like illness occurred in these persons within 1 week after culling. All 15 poultry workers had been working at their poultry farms for >6 years, and most of the animal health officials had experience in stamping out infected poultry. However, for the 36 temporary employees, previous contact histories with infected chickens were unknown. 

Throat swab specimens were collected from all contacts for virus detection within 7 days from the beginning of exposure to the virus, and paired serum samples were collected 21 days apart for serologic testing. Complete testing methods are described in the Technical Appendix. Participants were offered an inactivated influenza A(H5N1) vaccine on a voluntary basis on the day the first serum specimens were collected, and vaccination histories within 1 year before the specimen collection date were recorded through oral questionnaires. A total of 102 (72.3%) of the 141 participants were vaccinated: 22 (15.6%) received influenza A(H5N1) only; 39 (27.7%) received seasonal influenza vaccine only; 41 (29.0%) received both vaccines; and 39 (27.7%) received neither (Table 1). 

**Table 1:** HI antibody titers for influenza A(H5N2) virus in paired serum samples of 141 persons who had contact with infected poultry*

HI titers	Total	Second sample							Influenza vaccination history during previous 12 mo			
		<10	10	20	40	80	≥160		A(H5N1) only	Seasonal only	Both	None
First sample												
<10	13	0	6	7	0	0	0		0	4 (30.8)	7 (53.8)	2 (15.4)
10	57	2	4	48	3	0	0		16 (28.1)	5 (8.8)	22 (38.6)	14 (24.5)
20	32	0	5	11	15	1*	0		3 (9.4)	17 (53.1)	3 (9.4)	9 (28.1)
40	39	0	1	10	22	4*	2*		3 (7.7)	13 (33.3)	9 (23.1)	14 (35.9)
80	0	0	0	0	0	0	0		0	0	0	0
≥160	0	0	0	0	0	0	0		0	0	0	0
Total	141	2	16	76	40	5	2		22 (15.6)	39 (27.7)	41 (29.0)	39 (27.7)

*Values are no. persons or no. (%) persons. Serum samples with hemagglutination inhibition (HI) titer >80 underwent microneutralization testing. Criteria for determining seroconversion in HI antibodies with regard to influenza A(H5N2) viruses: 1) a >4-fold increase in antibody titers in the paired serum samples; and 2) HI titer >80 for the second sample.

We found all swab specimens were negative for influenza viruses by real-time reverse transcription PCR. However, hemagglutination inhibition (HI) and/or microneutralization (MN) test results showed 7 persons had antibody titers >80 for subtype H5N2 virus; 6 of these persons showed seroconversion for the virus (Table 2). Elevated antibodies against subtype H3N2 or H5N1 viruses were detected in some of the 6 persons who showed seroconversion (Table 2), which suggests that positive results for subtype H5N2 could be the result of cross-reactive antibodies from previous influenza vaccinations or infections. All 6 persons who showed seroconversion for influenza A(H5N2) virus had received vaccinations for influenza A(H5N1) and seasonal influenza (Table 2). Further, persons who received both influenza vaccinations showed a significant (p = 0.001) geometric mean titer increase in HI antibody against influenza A(H5N2) virus in the second samples, whereas those who did not receive both vaccinations did not show a similar increase. This finding indicates these heterologous vaccinations may influence HI antibody titers against influenza A(H5N2) virus.

**Table 2:** Serologic test results and vaccination and occupational histories for persons who had high antibody titers against influenza A(H5N2) virus and contact with infected poultry, Taiwan, 2012*†

Cont. no.	Sample date	Tested antigens, by influenza subtype							NS1-ELISA titer			Date and type of influenza vaccination		Occupation
		H5N2			H5N1	H1N1_pdm09_	H3N2							
		HI	MN		HI				NS1-pA	NS1-pB		A(H5N1)	Seasonal	
**1**	Mar 3	40	**<20**		80	<10	10		**1.68**	**1.4**		2012 Mar 3, 30	NA	Poultry worker
	Mar 30	80	**230**		80	<10	10							
2	Mar 5	**40**	**160**		40	20	80		0.89	0.86		2012 Mar 6, 28	2011 Oct 8	Poultry worker
	Mar 28	**160**	**450**		80	20	80							
**3**	Mar 5	40	**20**		80	20	40		**1.70**	**1.34**		2012 Mar 5	2012 Jan 20	Animal health official
	Mar 27	80	**80**		80	20	40							
4	Mar 5	40	**<20**		40	10	40		1.13	1.09		2012 Mar 5, Apr 2	2012 Jan 20	Animal health official
	Apr 2	80	**80**		40	10	40							
5	Mar 6	40	20		40	<10	320		1.21	1.02		2012 Mar 6, Apr 2	2011 Nov 26	Temp. employee
	Apr 2	80	30		40	<10	160							
6	Mar 6	**20**	**<20**		40	<10	**160**		1.17	1.08		2012 Mar 6, Apr 2	2012 Mar 5	Temp. employee
	Apr 2	**80**	**160**		80	<10	**640**							
7	Mar 5	**40**	**<20**		**40**	<10	**40**		0.98	1.05		2012 Mar 5, Apr 2	2012 Mar 5	Temp. employee
	Apr 2	**320**	**1,280**		**160**	10	**640**							

*Cont., contact; HI, hemagglutination inhibition; MN, microneutralization; NS1, nonstructural protein 1; H1N1pdm09, pandemic influenza A(H1N1); NA, not applicable (did not receive vaccination); temp., temporary. †Boldface indicates a >4-fold rise in antibody titer by HI or MN tests and a positive anti-NS1 antibody response by NS1-ELISA test between the first and second samples of paired serum samples. A positive anti-NS1 antibody response by NS1-ELISA assay was defined as each paired serum sample having a test absorbance of the second sample 30% higher than that of its first sample (ratio >1.30).

To investigate whether the influenza A(H5N2) antibodies were elevated as a result of exposure to that virus or because of vaccination with heterologous influenza viruses, we determined antibody levels to influenza A(H5N2) nonstructural protein 1 (NS1) (*12*–*15*). The NS1 protein is not readily incorporated into virions used to make inactivated influenza vaccine, so a response to NS1 protein would indicate active influenza A(H5N2) infection. Paired serum samples were analyzed by ELISA plates coated with 2 peptides, NS1^36–48^ (LRRDQKSLRGRGS, NS1-pA) and NS1^204–225^ (RSSNENGGPPLTPKQKREMART, NS1-pB), synthesized on the basis of the NS1 protein sequences of influenza A(H5N2) virus (NS1-pA, A/chicken/Taiwan/1209/2003) and influenza A(H3N2) virus (NS1-pB, A/Taiwan/4055/2009), respectively. The NS1-pA of the 2012 influenza A(H5N2) outbreak strain has an S48N substitution that is not found in the 2003 strain. 

For controls, we simultaneously analyzed 3 groups of paired serum samples with seroconversion (data not shown): 1) samples from 7 ferrets infected with different influenza virus strains (H1N1, n = 3; H3N2, n = 1; H5N1 [A/Vietnam/1194/2004], n = 1; and A[H1N1]pdm09, n = 2); 2) samples from 8 persons infected with influenza A(H1N1)pdm09 virus; and 3) samples from 9 persons who received vaccinations against influenza A(H5N1) virus. The resulting NS1 antibody responses were plotted (Figure). Five (71.4%) of 7 ferrets showed positive NS1 response against NS1-pA and all against NS1-pB (Figure, panel A), which indicates that influenza virus infection can cause a measurable anti-NS1 response after virus challenge. For influenza virus–infected persons, 3 (37.5%) of 8 showed responses against NS1-pA and NS1-pB (Figure, panel B), but for vaccinated persons, 1 (11.1%) of 9 showed responses against NS1-pA and none against NS1-pB (Figure, panel C). These patterns suggest that anti-NS1 response elicited by natural infection is stronger than that induced by vaccination. 

**Figure:**
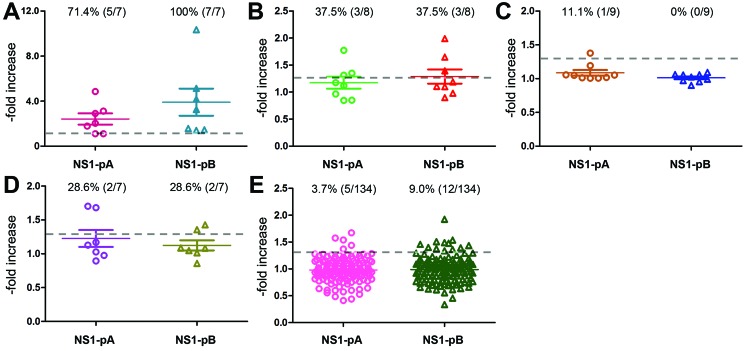
Antibody responses to 2 influenza A(H5N2) nonstructural protein 1 (NS1) peptides for paired serum samples from: A) influenza virus–infected ferrets; B) influenza virus–infected persons; C) influenza virus–vaccinated persons; D) persons in Taiwan who had contact with infected poultry during January–March 2012 and who showed seroconversion for influenza A(H5N2) virus exposure; and E) persons in Taiwan who had contact with infected poultry during January–March 2012 and who did not show seroconversion. Responses for each group were plotted by -fold increase from to second sample against NS1-pA (circles) and NS1-pB (triangles); numbers and percentages of positive responses for each sample set are indicated above each plot. Dashed lines indicate cutoff value for defining a positive response: results for the second sample in each pair 30% higher than those for first sample.

For the group of 7 contacts we identified who had elevated influenza A(H5N2) antibodies, 2 (contacts 1 and 3) had positive NS1 antibody response against both peptides; the remaining 5 did not (Figure, panel D). These results suggest that contact 1, a poultry worker, and contact 3, an animal health official, may have experienced recent influenza infections. 

To better establish the validity of using NS1 to distinguish infected from vaccinated persons, we analyzed paired serum samples for the 134 persons who did not show seroconversion for influenza A(H5N2) virus. Of these, 5 (3.7%) showed positive NS1 antibody response against NS1-pA and 12 (9.0%) against NS1-pB (Figure, panel E). This result suggests that an NS1-ELISA should not be used alone to determine influenza infection but can provide additional data to validate the results of protein-based serologic assays.

## Conclusions

In this study, we sampled 141 persons exposed to poultry infected with influenza A(H5N2) virus to assess virus shedding and used multiple serologic assays (including a novel NS1 ELISA) to determine seroconversion status. We found that 6 (4.3%) persons had elevated HA antibodies detected by HI and/or MN assays; a lower percentage (1.4%, 2/141) of subclinical infections was suspected after validation by NS1 antibody assays. The NS1-peptide B was designed on the basis of influenza A(H3N2) virus; however, it also reacted with antibodies elicited by viruses of different subtypes, which suggests that consensus residues may play an essential role in forming the epitope of NS1 protein. 

Our study has limitations. Patient histories of exposure to avian influenza viruses and influenza vaccination were given orally and thus may not be accurate, and mismatching between circulating viruses and antigens used in the study may have occurred. Also, recent seasonal influenza infection may interfere with the determination of subclinical infection with influenza A(H5N2) virus because the NS1 protein is remarkably conserved in type A influenza viruses. 

Cross-reactive antibodies in humans elicited from heterologous influenza viruses can complicate serologic, HA-based identification of influenza subtype. The NS1-ELISA method we describe may help determine the type more readily and improve diagnosis of subclinical infection in humans. Further, our findings indicate that occupational exposure to infected poultry may pose a risk for infection in humans.

Technical Appendix Expanded materials and methods for study of influenza A(H5N2) virus antibody seroconversion in humans after contact with infected poultry, Taiwan, 2012.
