# Supplementary material for: Influenza A(H5N2) Virus Antibodies in Humans after Contact with Infected Poultry, Taiwan, 2012
Source: Emerg Infect Dis. 2014 May;20(5):857–60. doi: 10.3201/eid2005.131393 (PMC4012807; doi:10.3201/eid2005.131393)
Supplement: Technical Appendix — Expanded materials and methods for study of influenza A(H5N2) virus antibody seroconversion in humans after contact with infected poultry, Taiwan, 2012. [file 13-1393-Techapp-s1.pdf]

Article DOI: http://dx.doi.org/10.3201/eid2005.131393

Influenza A(H5N2) Virus Antibodies in Humans after Contact with Infected Poultry, Taiwan, 2012

Technical Appendix

Materials

Viruses

Influenza A virus strains of different subtypes, including A/Chicken/Taiwan/1209/2003 (H5N2), A/Vietnam/1194/2004 (RG14, H5N1), A/California/07/2009 (H1N1pdm09) and A/Taiwan/4055/2009 (H3N2) were used for HI tests as antigens to investigate the existence of specific antibodies and the 2003 H5N2 isolate was also used for MN tests.

Vaccine

An inactivated H5N1 influenza vaccine (Aflunov®, Novartis Vaccines and Diagnostics, Siena, Italy) was used for the study subjects on a voluntary basis. The vaccine contains purified HA and NA surface antigens from influenza A/Vietnam/1194/2004 (H5N1) vaccine strain. Current circulating Asian H5N1 viruses have evolved into various clades with antigenic differences and are antigenically different from the vaccine strain.

Methods

Real-time RT-PCR

RNA was extracted from throat swab specimens by MagNa Pure LC extraction system (Roche). The extracted RNA was tested for influenza viruses by real-time RT-PCR (*1*). Briefly, this assay includes primers and probe sets to detect the M genes from all influenza A viruses and the HA genes of H, H3, and H5 subtypes

Hemaggutination inhibition (HI) test

Human sera were treated with receptor destroying enzyme (Denka Seiken, Japan) to remove non-specific hemagglutinins. Final concentrations of 1% horse (for H5N1 and H5N2) and 0.75% guinea pig erythrocytes (for H1N1pdm09 and H3N2) were used for HI tests (*2*). HI titers were expressed as the reciprocal of the highest dilution of serum that inhibited virus-induced hemagglutination.

Microneutralization (MN) test

MDCK cells were seeded in 96-well plates at the concentration of 1.5×10^4 cells/well. Two-fold serially diluted serum specimens were mixed with an equal volume of virus inoculums (100 $TCID_{50}$), followed by 1 hour of incubation at 37°C. After incubation, the mixture was added to confluent MDCK monolayers. Cells were cultured for 72 hours before the examination of cytopathic effect (CPE). The absence of CPE in individual wells was defined as protection. The assay was performed in quadruplicate.

NS1-ELISA test

An ELISA test for nonstructural protein 1 (NS1) antibody detection was developed in the study. Peptides of $NS1^{36\text{-}48}$ (LRRDQKSLRGRGS, designated as peptide A), $NS1^{161–175}$

(SPLPSLPGHTDEDVK, peptide B) were used in the test. The peptide A was synthesized according to the previous study (*3*) with modifications to match that of the 2003 H5N2 virus and the peptide B was newly designed in this study and predicted by using Antibody Epitope Prediction in IEDB Analysis Resource (http://tools.immuneepitope.org/tools/bcell/iedb_input). ELISA plates were coated overnight at 4°C with each peptide (1 μg/well) diluted in carbonate-bicarbonate buffer (pH 9.6), followed by blocking with PBSB buffer (PBS with 1% bovine serum albumin). Serum specimens were heat inactivated at 56 °C for 30 minutes and then diluted 1:100 with PBSTB buffer (PBST with 1% bovine serum albumin). Serum antibodies bound to the coated peptides were detected by using horseradish peroxidase-labeled goat anti-human IgG antibodies (KPL, USA). After adding the TMB/E substrate (Millipore, USA) for 15-30 minutes, sulfuric acid was added to stop the reaction. The absorbance at 450 nm was measured and analyzed. Wells that coated with 1% BSA were used for controls. The normalized absorbance measurement of each serum specimen was calculated by using OD_{450nm} value of the peptide-coated well minus that of BSA-coated well. For each paired serum, if the value of the second sample was 30% higher than that of its first sample, it was defined as a positive anti-NS1 antibody response.

References

1. Yang JR, Lo J, Liu JL, Lin CH, Ho YL, Chen CJ, et al. Rapid SYBR green I and modified probe real-time reverse transcription-PCR assays identify influenza H1N1 viruses and distinguish between pandemic and seasonal strains. J Clin Microbiol. 2009;47:3714–6. PubMed http://dx.doi.org/10.1128/JCM.01646-09

2. World Health Organization. Manual for the laboratory diagnosis and virological surveillance of influenza [cited 2013 Feb 8]. http://whqlibdoc.who.int/publications/2011/9789241548090_eng.pdf

3. Tumpey TM, Alvarez R, Swayne DE, Suarez DL. Diagnostic approach for differentiating infected from vaccinated poultry on the basis of antibodies to NS1, the nonstructural protein of influenza A virus. J Clin Microbiol. 2005;43:676–83. PubMed http://dx.doi.org/10.1128/JCM.43.2.676-683.2005
